# Synthesis of Magnetite Nanoparticles through a Lab-On-Chip Device

**DOI:** 10.3390/ma14195906

**Published:** 2021-10-08

**Authors:** Cristina Chircov, Alexandra Cătălina Bîrcă, Alexandru Mihai Grumezescu, Bogdan Stefan Vasile, Ovidiu Oprea, Adrian Ionuț Nicoară, Chih-Hui Yang, Keng-Shiang Huang, Ecaterina Andronescu

**Affiliations:** 1 Department of Science and Engineering of Oxide Materials and Nanomaterials, University Politehnica of Bucharest, 011061 Bucharest, Romania; cristina.chircov@yahoo.com (C.C.); ada_birca@yahoo.com (A.C.B.); bogdan.vasile@upb.ro (B.S.V.); adi.nicoara18@gmail.com (A.I.N.); ecaterina.andronescu@upb.ro (E.A.); 2 Research Institute of the University of Bucharest—ICUB, University of Bucharest, 050657 Bucharest, Romania; 3 Academy of Romanian Scientists, 54 Spl. Independentei, 050045 Bucharest, Romania; 4 Department of Inorganic Chemistry, Physical Chemistry and Electrochemistry, University Politehnica of Bucharest, 1-7 Polizu St., 011061 Bucharest, Romania; ovidiu73@yahoo.com; 5 Department of Biological Science and Technology, I-Shou University, Kaohsiung 824, Taiwan; chyang@isu.edu.tw; 6 Pharmacy Department of E-Da Hospital, Kaohsiung 824, Taiwan; 7 Taiwan Instrument Research Institute, National Applied Research Laboratories, Hsinchu 300, Taiwan; 8 The School of Chinese Medicine for Post-Baccalaureate, I-Shou University, Kaohsiung 840301, Taiwan; huangks@isu.edu.tw

**Keywords:** magnetite nanoparticles, iron oxide nanoparticles, microfluidics, lab-on-chip

## Abstract

Magnetite nanoparticles (MNPs) represent one of the most intensively studied types of iron oxide nanoparticles in various fields, including biomedicine, pharmaceutics, bioengineering, and industry. Since their properties in terms of size, shape, and surface charge significantly affects their efficiency towards the envisaged application, it is fundamentally important to develop a new synthesis route that allows for the control and modulation of the nanoparticle features. In this context, the aim of the present study was to develop a new method for the synthesis of MNPs. Specifically, a microfluidic lab-on-chip (LoC) device was used to obtain MNPs with controlled properties. The study investigated the influence of iron precursor solution concentration and flowed onto the final properties of the nanomaterials. The synthesized MNPs were characterized in terms of size, morphology, structure, composition, and stability. Results proved the formation of magnetite as a single mineral phase. Moreover, the uniform spherical shape and narrow size distribution were demonstrated. Optimal characteristics regarding MNPs crystallinity, uniformity, and thermal stability were obtained at higher concentrations and lower flows. In this manner, the potential of the LoC device is a promising tool for the synthesis of nanomaterials by ensuring the necessary uniformity for all final applications.

## 1. Introduction

Iron oxide nanoparticles, including magnetite (Fe_3_O_4_), maghemite (γ-Fe_2_O_3_), hematite (α-Fe_2_O_3_), and mixed ferrites (MFe_2_O_4_, where M = Co, Mn, Ni or Zn), belong to the class of ferrimagnetic materials [1,2,3,4]. Magnetite is one of the most commonly studied and utilized types of naturally occurring iron oxide [5,6,7], characterized by a crystalline cubic inverse spinel structure and an Fd3m space group with an 8.394 Å cell parameter. Moreover, the 32 O_2_^−^ ions form a face-centered cubic closed packing structure, where ferrous ions are disposed in half of the octahedral lattice sites and ferric ions are disposed of in the other half of the octahedral lattice sites and the tetrahedral lattice sites [5,6,8,9].

Since they can be synthesized in variety of sizes and shapes and further manipulated through external magnetic fields, magnetite nanoparticles (MNPs) can be employed in magnetic resonance imaging and magnetic particle imaging as imaging contrast enhance ment agents; targeted delivery of bioactive molecules, drugs, antibodies, nucleic acids, and proteins; hyperthermia for cancer treatment; cell separation and detection; biosensing and immunoassays; tissue repair; and body fluid detoxification [1,8,9,10,11]. Additionally, MNPs have demonstrated their potential in oil recovery, catalysis, and water remediation applications [2]. Therefore, MNPs represent promising alternatives to conventional methods in various applications within the biomedical, pharmaceutical, bioengineering, and industrial fields.

Size, shape, and surface chemistry are the key characteristics that influence the biodistribution, pharmacokinetics, body clearance, and toxicity of MNPs [12,13,14]. As the saturation magnetization decreases with size, MNPs are categorized into superparamagnetic iron oxide nanoparticles, ultrasmall superparamagnetic iron oxide nanoparticles, and monocrystalline iron oxide nanoparticles, a subset of ultrasmall superparamagnetic iron oxide nanoparticles. Therefore, the size represents a fundamental factor that determines the magnetic properties and the consequent response to magnetic fields of MNPs [13,15]. Furthermore, within the biomedical field, size influences the filtration, blood circulation time, and cellular uptake of MNPs [12,13,16,17]. Additionally, multiple studies have demonstrated a direct relation between MNPs properties and their toxicity [18,19].

In this context, the synthesis method is a fundamentally important choice, as any variation in the process can consequently result in significant changes of the MNPs final properties (12). Conventional methods that have been widely employed include chemical routes, with co-precipitation as the most common, physical techniques, and biological or “green” methods [3,5,8,20,21]. However, novel and more precise methods have recently gained increased scientific interest. Microfluidic methods are currently emerging as promising techniques for synthesizing MNPs with controlled and tailorable size, shape, and surface chemistry [5]. Microfluidics is the field that encompasses principles from chemistry, fluid dynamics, and material science, and allows for the accurate manipulation of small volumes through the use of microchannels. In this manner, more efficient mixing, shorter residence times, and superior final products are obtained [5,22].

In this context, the present paper aimed to develop a new microfluidic method for the synthesis of MNPs with uniform properties. Specifically, a lab-on-chip (LoC) device was employed for studying the influence of iron precursor solution concentrations and flows onto the final properties of the nanoparticles.

## 2. Materials and Methods

### 2.1. Materials

Ferrous sulfate heptahydrate (FeSO_4_·7H_2_O), ferric chloride (FeCl_3_), and sodium hydroxide (NaOH) were purchased from Sigma-Aldrich. All chemicals were of analytical purity and used with no further purification.

### 2.2. Methods

#### 2.2.1. LoC Device Fabrication

**Figure 1:**
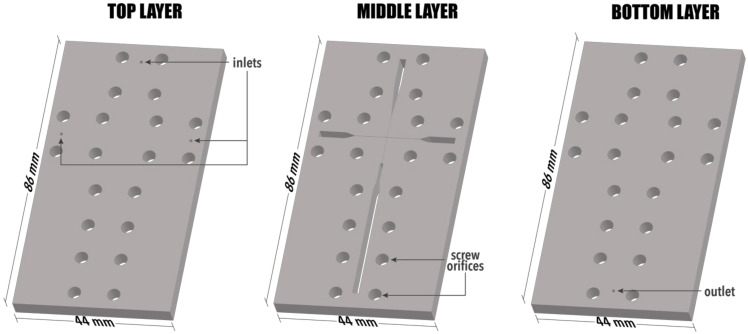
Configuration of the three layers comprising the LoC device.

**Figure 2:**
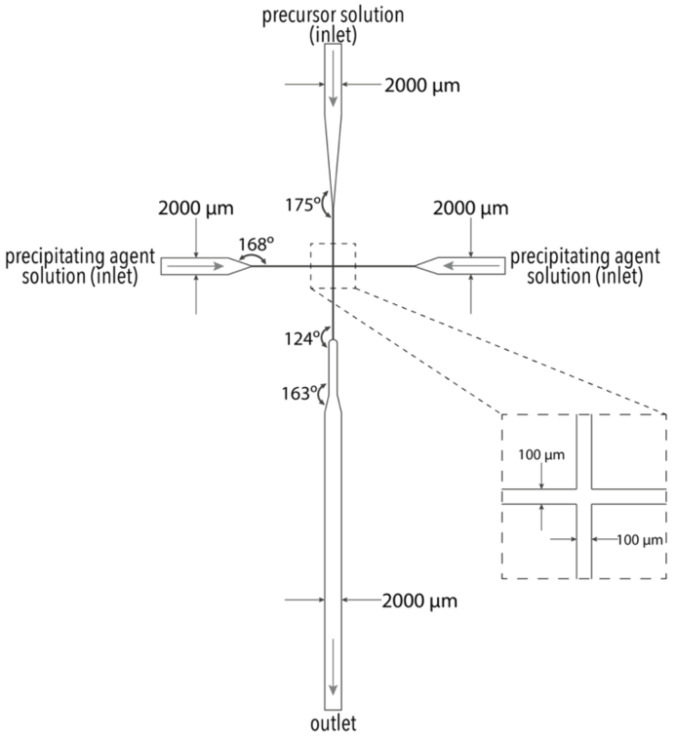
Configuration of the cross-junction channel within the middle layer.

The LoC device was constructed using a CO_2_ laser machine (LaserPro Venus, GCC, New Taipei, Taiwan) on poly(methyl methacrylate) (PMMA) substrate, according to previous studies [23,24,25,26]. The LoC device consists of three layers of the same size (i.e., length × width × depth = 86 mm × 44 mm × 1 mm) (Figure 1). The top cover layer contains three reagent inlets and 20 screw orifices for fastening, the middle layer, a cross-junction channel (Figure 2), and 20 screw orifices, and the bottom layer, a reaction product outlet, and 20 screw orifices. The three layers were combined by 20 M4 bolts (0.5 mm pitch, 4.0 mm in diameter, and tightened by a force of 2 Nm) to construct the microfluidic chip. Additionally, PMMA pads were used at each inlet and outlet for fastening the Teflon tubes (0.76 mm inner diameter and 1.22 mm outer diameter) used for solution injection and product collection.

#### 2.2.2. Standard Solution Preparation

The stock precursor solutions with three different concentrations were prepared by dissolution FeSO_4_·7H_2_O and FeCl_3_ in distilled water under continuous magnetic stirring. After complete homogenization, the solutions were transferred to 1000 mL volumetric flasks, and distilled water was added to reach the final volume.

The precipitating agent solution was prepared by dissolving NaOH 1M in 1000 mL distilled water.

#### 2.2.3. MNPs Synthesis

MNPs were prepared through the co-precipitation method using the LoC device. The precursor and NaOH 1 M solutions were simultaneously injected into the LoC device through the Teflon tubes using two automated syringe pumps. Specifically, the precursor solutions were introduced into the middle channel through the central inlet at three different flows, while the precipitating agent solution was injected from the two side inlets at 150 mL/h for each channel. The so-obtained nanoparticle dispersions were dripped from the outlet, collected, and washed with distilled water using a high-power permanent magnet for nanoparticle separation until a neutral pH value. Subsequently, the nanoparticles were dried at 40 °C for 48 h.

In this manner, nine types of MNPs samples were obtained and coded as C1_20, C1_40, C1_60, C2_20, C2_40, C2_60, C3_20, C3_40, and C3_60 (Table 1).

### 2.3. Physicochemical Characterization

#### 2.3.1. LoC Device Characterization

To assess its surface morphology at the contact with the reaction solutions, the LoC device was characterized before and after nanoparticle synthesis through Scanning Electron Microscopy (SEM) with a microscope purchased from Thermo Fisher—former FEI (Eindhoven, The Netherlands). The obtained images were produced by recording the resultant secondary electron beam with an energy of 30 keV.

#### 2.3.2. MNPs Characterization

##### X-ray Diffraction (XRD)

Grazing incidence XRD (GIXRD) was performed with a PANalytical Empyrean diffractometer (PANalytical, Almelo, The Netherlands), using the CuK_α_ radiation (1.541874 Å) equipped with a 2 × Ge (2 2 0) hybrid monochromator for Cu and a parallel plate collimator on the PIXcel3D. Scanning was performed on the 2θ axis in the range of 5–80° with an incidence angle of 0.5° with a step size of 0.04°, and the time for each step was 3 s.

##### Infrared (IR) Spectroscopy

IR spectra were obtained with a Nicolet iN10 MX Fourier transform (FT)-IR microscope (Thermo Fischer Scientific, Waltham, MA, USA) with a liquid nitrogen-cooled mercury cadmium telluride detector with the measurement range between 4000 and 700 cm^−1^. Spectra collection was performed in reflection mode at a resolution of 4 cm^−1^. For each spectrum, 32 scans were co-added and converted to absorbance using the OmincPicta software (Thermo Scientific).

##### SEM. Energy Dispersive X-ray Analysis (EDX)

To investigate the morphology and size of the MNPs, the samples were placed in the analysis chamber of the scanning electron microscope coupled with an energy dispersive spectrometer. The obtained images are produced by recording the resultant secondary electron beam with an energy of 30 keV.

##### Transmission Electron Microscopy (TEM). High-Resolution TEM (HR-TEM). Selected Area Electron Diffraction (SAED)

The samples were placed on a carbon-coated copper grid at room temperature and analyzed using a high-resolution Tecnai^TM^ G2 F30 S-TWIN transmission microscope equipped with SAED, purchased from the FEI (Hillsboro, OR, USA). The microscope operates in transmission mode at a 300 kV voltage, the point and line resolution guaranteed with the values of 2 Å and 1 Å, respectively. The granulometric distribution was assessed by creating histograms corresponding to the TEM images using the ImageJ software, measuring approximately 100 particles for each sample.

##### Differential Scanning Calorimetry and Thermogravimetry (DSC-TG)

Thermogravimetric analysis was performed using an STA TG/DSC Netzsch Jupiter 449 °C equipment (Selb, Germany). The temperature range was between 25 and 900 °C in a dynamic atmosphere of 50 mL/min air with a heating rate of 10 K/min in an alumina crucible.

##### Dynamic Light Scattering (DLS). Zeta Potential

The hydrodynamic diameter and the surface charge of the MNPs were obtained using the DLS technique (DelsaMax Pro, Backman Coulter, Brea, CA, USA). The nanoparticles were dispersed using an ultrasonication bath into deionized water (pH ~ 6.9) at similar concentrations.

## 3. Results and Discussion

**Figure 3:**
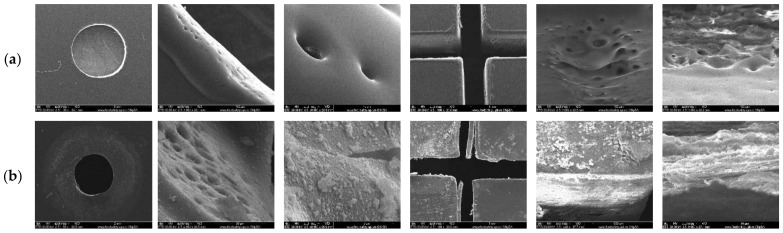
SEM images of the outlet and the cross-junction area of the microchannel before (**a**) and after (**b**) utilization.

The surface morphology of the microchannel within the LoC device was assessed both before and after the synthesis of the MNPs through SEM. As can be seen in Figure 3, the LoC device is characterized by surface defects at the laser cutting areas associated with the fabrication method. The presence of such defects could further promote the accumulation of the nanoparticles, thus forming submicronic nanoparticle aggregates. Furthermore, the results show a considerable deterioration of the device after repeated syntheses, demonstrating a limit of ten experiments that can be performed on the same microfluidic chip.

Other studies reported in the literature implement different microchannel configurations for the synthesis of nanoparticles. For instance, Rao et al. investigated a microfluidic chip consisting of two inlets, an outlet, a Y-shaped merging channel, an S-shaped mixing channel, and an electroporation zone to synthesize erythrocyte membrane-coated magnetic nanoparticles [27]. Generally, MNPs-based microfluidic approaches focus on developing highly accurate sensors and biosensors, using MNPs synthesized through conventional methods [28,29,30,31]. Therefore, the novelty of our work is demonstrated by the currently limited available literature employing microfluidic approaches for MNPs synthesis.

**Figure 4:**
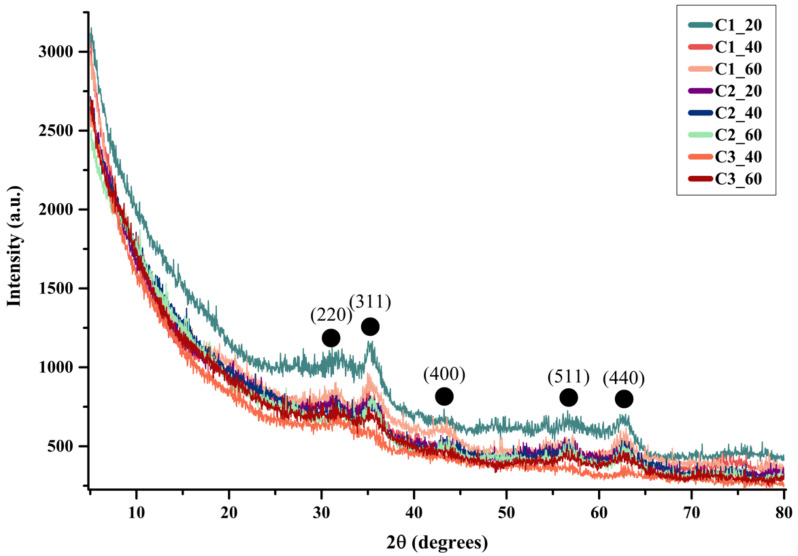
Diffractograms for the MNPs samples (●—Fe_3_O_4_).

The crystallinity of the MNPs was evaluated through XRD (Figure 4). As can be seen from the diffractograms, a single crystalline phase with diffractive interferences characteristic for iron oxide (Fe_3_O_4_) in the Fd-3m cubic crystal system and the associated Miller indices (according to the PDF 04-011-5952 [32]) was observed in all samples. It can be seen that the values of the diffraction peak intensities and, consequently, the crystallinity of the samples, decrease when the concentration of the precursor solution is decreased, and the flow is increased. Thereby, the C1_20 sample appeared to be the most crystalline.

Furthermore, the samples were subjected to Rietveld refinement using the HighScore Plus software (version 3.0, PANalytical, Almelo, The Netherlands) in order to determine the crystallite size and the crystallinity of the nanoparticles. Thus, Table 2 reveals the average crystallite size and the calculated crystallinity of the samples. Additionally, the analysis showed the presence of magnetite in a 100% proportion, thus proving the purity of the samples. Therefore, it is safe to assume that the optimal parameters for MNPs synthesis using the proposed LoC device were established, involving higher precursor solution concentrations and lower fluid flows. Future studies should start from the C1 concentration and further increase it, while concomitantly decreasing the flow below 20 mL/h.

As previously observed, the crystallinity of the samples decreases with the increase of the flow and the decrease of the precursor solution concentration. Since it is a well-known fact that the smaller the crystallite size, the lower the crystallinity of the powder is, the reduced crystallinity values that were calculated are in accordance with the available literature [33]. As can be seen, the crystallite size of the obtained MNPs is considerably reduced, in the range of 1.4 to 3.6 nm, which could explain the low crystallinity values.

**Figure 5:**
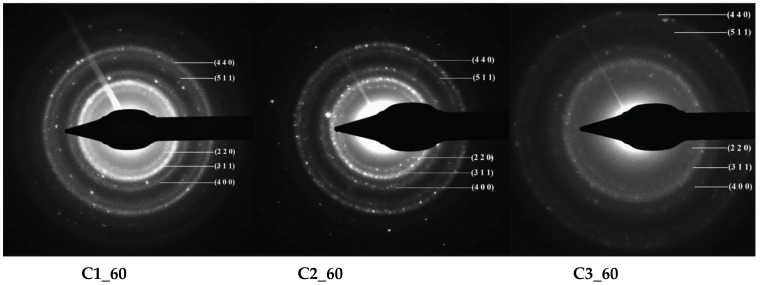
SAED diffraction patterns and the corresponding Miller indices for the MNPs samples.

The crystallinity of the synthesized nanoparticles was also assessed through SAED analysis (Figure 5). In this manner, the XRD results regarding the presence of iron oxide as a single crystalline phase were further confirmed through the diffraction patterns and the corresponding Miller indices. Moreover, a decrease in the concentration of the iron precursor solutions leads to a decrease in the nanoparticle crystallinity, which can be seen through more diffused diffraction rings [34,35]. In this manner, the presence of the amorphous iron oxide-hydroxide in the C3 samples was confirmed.

**Figure 6:**
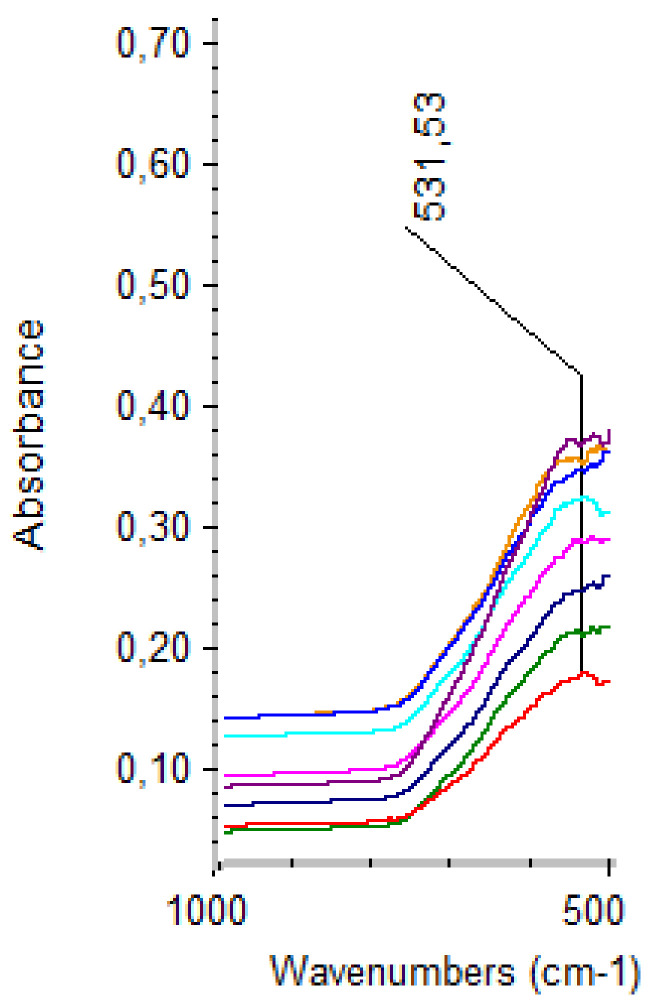
FT-IR spectra for the MNPs samples.

The functional groups present within the synthesized samples were assessed through FT-IR. In this context, as can be seen in Figure 6, the formation of magnetite is confirmed by the absorption band at 531.53 cm^−1^ characteristics to the Fe-O vibration in magnetite [36,37,38,39].

**Figure 7:**
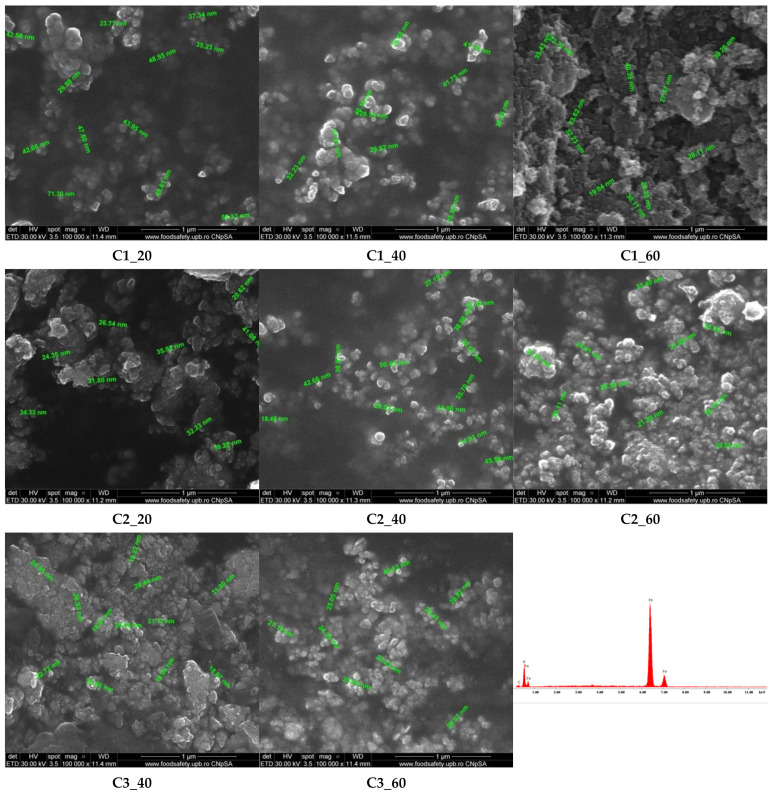
SEM images and EDX spectrum for the MNPs synthesized through the LoC device.

The morphological and structural properties of the MNPs synthesized through the LoC device were evaluated through SEM, TEM, and HR-TEM, whereas the elemental composition was determined through EDX. In this context, SEM images reveal a tendency of nanoparticle agglomeration in all samples (Figure 7). Moreover, it can be observed that while a uniform spherical shape characterizes all samples, there are several differences in terms of particle dimension, ranging from 20 to 50 nm. Precisely, the C1_20 sample contains the largest nanoparticles, while the C2_20 and C3_40, the smallest nanoparticles. Therefore, it can be concluded that nanoparticle size variations can be reduced by decreasing the precursor solution concentration and the flow. The studies available in the literature involving the use of microchannel-based syntheses of MNPs showed smaller nanoparticle sizes, ranging between 4 and 7 nm [40,41]. The reduced size could be the result of the reduced microchannel diameter size, which was around 20 μm. Furthermore, a study by Bemetz et al. implemented a similar microfluidic approach and reported a primary particle size of about 25 nm. Additionally, they observed that an increase in the concentration of the precipitating agent solution leads to reduced particle sizes [42]. The only elements identified within the samples are Fe and O, as C is generally attributed to the substrate, thus confirming the purity of the samples (Figure 7).

**Figure 8:**
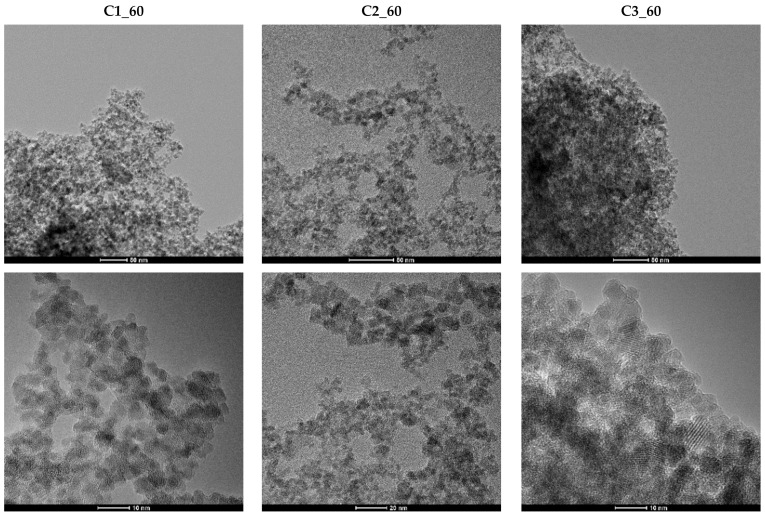

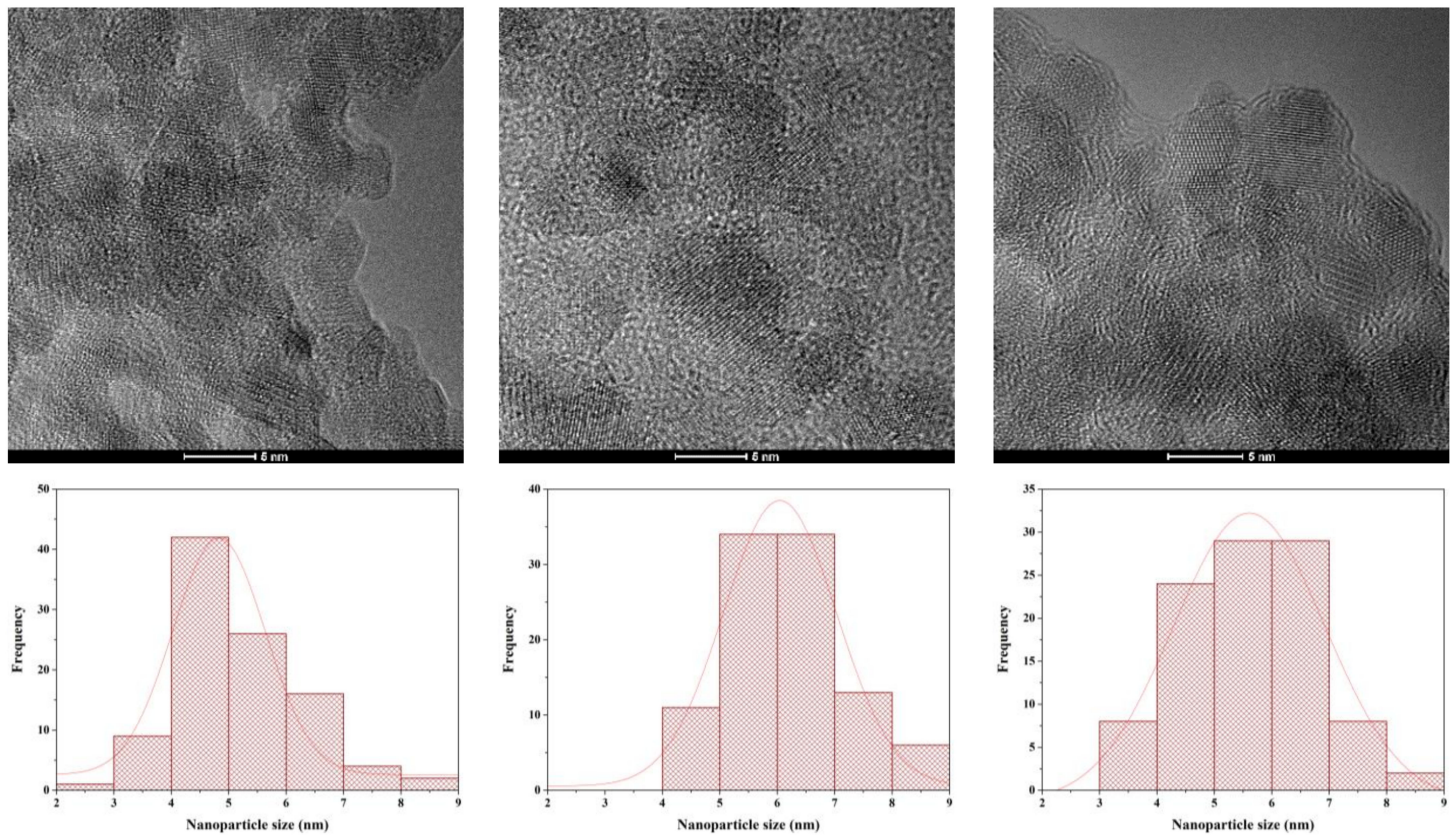
TEM images and the corresponding histograms for samples C1_60, C2_60, and C3_60.

The SEM observations were further confirmed by TEM and HR-TEM (Figure 8). In this context, the synthesized nanoparticles are characterized by a uniformly spherical shape and agglomeration tendency. However, the histograms created using TEM images reveal a considerably narrow granulometric distribution, with nanoparticle sizes of less than 10 nm, especially for the C1 samples (Figure 8). The Gaussian fitting of the histograms revealed an average nanoparticle size of 4.82 nm (R^2^ of 0.91, width (w) = 1.61) for C1_60, 6.04 nm (R^2^ of 0.98, w = 1.97) for C2_60, and 5.60 nm (R^2^ of 0.96, w = 2.64) for C3_60. The R^2^ values that were obtained (close to 1) prove the limited variability around the mean values and, consequently, the uniformity of the nanoparticle sizes. In this manner, as compared to the previously mentioned studies, it can be said that although the present LoC device contains larger microchannels, it allows for similarly small nanoparticles [40,41]. Furthermore, compared to other studies synthesizing magnetite nanoparticles through other methods, such as chemical co-precipitation [43,44,45], the microfluidic method involved in this study led to significantly narrower size distributions. In this manner, the uniformity of the synthesized nanoparticles and the efficiency of the LoC device was demonstrated.

**Figure 9:**
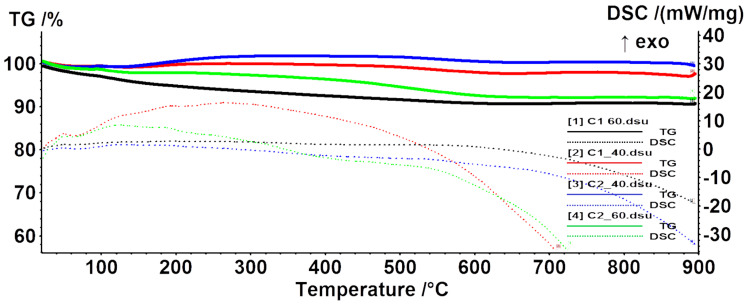
DSC-TG graphs for the synthesized MNPs.

The thermal stability of the nanoparticles was assessed through DSC-TG (Figure 9). As can be observed, the mass loss is proportional to the concentration of the precursor solution and inversely proportional to the flow. The reason behind this behavior could be related to a higher amount of hydroxyl groups present on the surface. As the mass loss at lower temperatures is usually related to the adsorbed water molecules and –OH moieties, we can conclude that the samples obtained with a flow of 60 mL/h have the highest amount of water adsorbed. In addition, it can be observed that the samples obtained at 40 mL/h presents a mass increase after 200 °C. This indicates the existence of a higher proportion of Fe^2+^ ions, which can be oxidized to Fe^3+^, hence the mass increase. In other words, the Cx_40 samples are more prone to oxidation from magnetite to maghemite [46]. Nevertheless, the red, nonmagnetic residual powder obtained at 900 °C is most probably hematite [47].

DLS was utilized to determine the hydrodynamic diameter and the zeta potential for the synthesized MNPs. In this context, Table 3 specifies the hydrodynamic diameter distribution for all samples. It can be observed that the uniformity of nanoparticle sizes is further confirmed, as more than 95% of the scanned particles have the same diameter. Thus, the size of MNPs, as shown by the hydrodynamic diameter of MNPs follows a monomodal distribution [48], which further confirms the TEM results. However, as there is a significant difference between the dry and wet surface chemistry of MNPs, the nanoparticle size ranges from TEM and DLS measurements differ substantially [49].

Moreover, the obtained results reveal a tendency of size decrease inversely proportional to the flow. The results cannot be compared to those reported by Bemetz et al. since their experiment involved an additional step of alendronate coating, which modifies the surface chemistry of the nanoparticles and the interactions with the solvent [42].

Furthermore, the zeta potential values (Table 4) confirm the tendency of nanoparticle agglomeration. The lowest values were registered for the C3 samples, thus demonstrating the highest stability due to the high amount of negative charges onto the surface of the nanoparticles [50]. Generally, low (close to zero) zeta potential values are associated with a lack of repelling forces and consequently to limited dispersion stability [51]. In this context, adding a functionalization step within the synthesis process is necessary to improve the stability of the MNPs [52].

In this manner, the proposed LoC device allows for the synthesis of MNPs with uniform properties. Specifically, the nanoparticle size distribution obtained for each type of the samples is considerably narrow, thus allowing for a standardized and controlled synthesis in regard to the uniformity of the samples.

## 4. Conclusions

The synthesis of MNPs through conventional methods, such as the co-precipitation of ferrous and ferric ions, generally leads to nanoparticle properties variations. Specifically, such methods lack the possibility of synthesizing highly uniform nanoparticles in terms of size, shape, and crystallinity. Thus, this study aimed to apply an LoC device for the synthesis of highly uniform MNPs. The influence of precursor concentration and flow was investigated through XRD, SEM, EDX, TEM, HR-TEM, SAED, FT-IR, DSC-TG, DLS, and zeta potential. The results demonstrated that the size and size distribution, crystallinity, and stability of the nanoparticles could be modulated by modifying the concentration of the precursor solution and the flow of the experiment. Therefore, the use of the LoC device for the synthesis of MNPs allows for a highly controlled environment for the co-precipitation process, precise modulation of the nanoparticle properties, and the synthesis of highly uniform nanoparticle characteristics.

## Figures and Tables

**Table 1:** The concentration of Fe^2+^ and Fe^3+^ and the flow of the precursor solution for each sample.

Sample Code	C1_20	C1_40	C1_60	C2_20	C2_40	C2_60	C3_20	C3_40	C3_60
**FeSO_4_·7H_2_O concentration [%]**	0.5	0.5	0.5	0.3	0.3	0.3	0.1	0.1	0.1
**FeCl_3_ concentration [%]**	0.8	0.8	0.8	0.48	0.48	0.48	0.16	0.16	0.16
**Flow [mL/h]**	20	40	60	20	40	60	20	40	60

**Table 2:** The average crystallite size and he crystallinity of the MNPs samples.

Sample	C1_20	C1_40	C1_60	C2_20	C2_40	C2_60	C3_40	C3_60
**Average crystallite size [Å]**	26.21	20.96	20.99	22.98	30.01	36.17	14.52	16.70
**Standard deviation**	3.45	1.65	1.41	2.80	3.63	5.82	0.58	0.56
**Crystallinity [%]**	12.11	10.58	9.99	9.81	5.30	6.39	5.27	9.05

**Table 3:** The hydrodynamic diameters for the MNPs samples.

Sample	Hydrodynamic Diameter [nm]	% Mass
**C1_20**	26.7	0.0
	811.6	2.2
	4403.1	97.8
**C1_40**	126.3	0.2
	631.3	3
	4492.3	96.8
**C1_60**	68.7	0.4
	765.3	33.3
	4772.7	66.3
**C2_20**	100.2	0.2
	2966.2	99.8
**C2_40**	244.4	0.2
	3322.9	99.8
**C2_60**	143.7	1.3
	4628.8	98.7
**C3_20**	94.6	0.8
	918.1	99.2
**C3_40**	91.5	0.2
	2051.2	99.8
**C3_60**	130.4	2.1
	760.8	13.6
	4459.4	84.3

**Table 4:** Zeta potential values for the synthesized MNPs.

Sample	C1_20	C1_40	C1_60	C2_20	C2_40	C2_60	C3_20	C3_40	C3_60
**Zeta potential [mV]**	−7.77	−12.78	−4.87	−6.62	−10.41	−9.39	−69.51	−72.54	−39.26

## Data Availability

Not applicable.
